# French 2013 guidelines for antiretroviral therapy of HIV-1 infection in adults

**DOI:** 10.7448/IAS.17.1.19034

**Published:** 2014-06-17

**Authors:** Bruno Hoen, Fabrice Bonnet, Constance Delaugerre, Pierre Delobel, Cécile Goujard, Marianne L’Hénaff, Renaud Persiaux, David Rey, Christine Rouzioux, Anne-Marie Taburet, Philippe Morlat, on behalf of the 2013 French HIV expert group

**Affiliations:** 1 Service de maladies infectieuses, Centre Hospitalier Universitaire, Pointe-à Pitre, France; 2 Service de médecine interne et maladies infectieuses, Centre Hospitalier Universitaire, Bordeaux, France; 3 Laboratoire de virologie, Hôpital Saint Louis, Assistance Publique des hôpitaux de Paris, Paris, France; 4 Service des maladies infectieuses et tropicales, Centre Hospitalier Universitaire, Toulouse, France; 5 Service de médecine interne, Hôpital du Kremlin-Bicêtre, Assistance Publique des hôpitaux de Paris, Paris, France; 6 Groupe interassociatif traitement et recherche thérapeutique (TRT-5), Association ARCAT, Paris, France; 7 Groupe interassociatif traitement et recherche thérapeutique (TRT-5), Association AIDES, Paris, France; 8 Centre d’information et de soins de l’immunodéficience humaine, Hôpitaux universitaires, Strasbourg, France; 9 Laboratoire de virologie, Hôpital Necker-Enfants malades, Assistance Publique Hôpitaux de Paris, Paris, France; 10 Laboratoire de pharmacologie, Hôpital du Kremlin-Bicêtre, Assistance Publique Hôpitaux de Paris, Paris, France

**Keywords:** antiretroviral treatment, guidelines, first-line therapy, virologic failure, cost of treatment.

## Abstract

**Introduction:**

These guidelines are part of the French Experts’ recommendations for the management of people living with HIV/AIDS, which were made public and submitted to the French health authorities in September 2013. The objective was to provide updated recommendations for antiretroviral treatment (ART) of HIV-positive adults. Guidelines included the following topics: when to start, what to start, specific situations for the choice of the first session of antiretroviral therapy, optimization of antiretroviral therapy after virologic suppression, and management of virologic failure.

**Methods:**

Ten members of the French HIV 2013 expert group were responsible for guidelines on ART. They systematically reviewed the most recent literature. The chairman of the subgroup was responsible for drafting the guidelines, which were subsequently discussed within, and finalized by the whole expert group to obtain a consensus. Recommendations were graded for strength and level of evidence using predefined criteria. Economic considerations were part of the decision-making process for selecting preferred first-line options. Potential conflicts of interest were actively managed throughout the whole process.

**Results:**

ART should be initiated in any HIV-positive person, whatever his/her CD4 T-cell count, even when >500/mm^3^. The level of evidence of the individual benefit of ART in terms of mortality or progression to AIDS increases with decreasing CD4 cell count. Preferred initial regimens include two nucleoside reverse transcriptase inhibitors (tenofovir/emtricitabine or abacavir/lamivudine) plus a non-nucleoside reverse transcriptase inhibitor (efavirenz or rilpivirine), or a ritonavir-boosted protease inhibitor (atazanavir or darunavir). Raltegravir, lopinavir/r, and nevirapine are recommended as alternative third agents, with specific indications and restrictions. Specific situations such as HIV infection in women, primary HIV infection, severe immune suppression with or without identified opportunistic infection, and person who injects drugs are addressed. Options for optimization of ART once virologic suppression is achieved are discussed. Evaluation and management of virologic failure are described, the aim of any intervention in such situation being to reduce plasma viral load to <50 copies/ml.

**Conclusion:**

These guidelines recommend that any HIV-positive individual should be treated with ART. This recommendation was issued both for the patient’s own sake and for promoting treatment as prevention.

Guidelines presented in this article are part of the French Experts’ recommendations for the management of people living with HIV/AIDS, which were made public and submitted to the national health authorities in September 2013 [1].

In addition to establishing evidence-based recommendations, the experts kept in mind the following four factors that depict the French national context:

- there is no indication that the incidence of new HIV infections is decreasing,
- it has been estimated that up to 25% of the people living with HIV/AIDS (30,000 out of 120,000) are unaware of their condition and are the primary source of new infections,
- screening strategies and procedures should be altered so that a higher number of people living with HIV, especially those newly diagnosed, are linked to care,
- economic considerations are to be taken into account in a country where care for people living with HIV is provided for free and the cost is borne by the national budget.

## Methods

Ten members of the French HIV 2013 expert group were appointed to write the project of guidelines on antiretroviral therapy (ART). This subgroup included five clinicians, two virologists, one pharmacologist, and two patient representatives. They systematically reviewed the more recent literature. Data presented at scientific conferences were also considered. Two additional experts were questioned about specific fields of interest. The chairman of the subgroup was responsible for writing and harmonizing the guidelines. Each of the following specific sections was assigned to one of the subgroup members. All members of the subgroup met several times, either physically or by teleconference over a six-month period. The draft guidelines that resulted from this process were presented to, discussed within and finalized by the whole expert group over three in-person meetings to obtain a consensus. Prior to starting the work, each member of the plenary group and of the sub-group completed a public declaration of interest. Three members of the plenary group did not participate in the debate on ART due to potential conflict of interest. Most of the recommendations were graded for strength and level of evidence using the classification described in the tables below.

**Table:** Strength of recommendations

Scale	Definition
A	Strong evidence to support the recommendation
B	Moderate evidence to support the recommendation
C	Insufficient evidence to support the recommendation

**Table:** Level of evidence: type of data used in the recommendations*

Scale	Definition
I	Evidence from at least one randomized, controlled clinical trial; meta-analyses of randomized trials
II	Evidence from non-randomized clinical trials, cohort or case-control studies; meta-analyses of cohort or case-control studies
III	Recommendation based on the panel’s analysis of other available data

* We only considered data published in peer-reviewed literature

## When to start?

### Asymptomatic patients

#### Morbidity and mortality benefits

Several cohort studies [2–5] have shown that initiation of ART when the CD4 T-cell count is between 350 and 500/mm^3^, rather than <350/mm^3^, is beneficial in terms of mortality and/or progression to AIDS. For ART initiation at a CD4 T-cell count >500/mm^3^, the clinical benefit remains controversial [2,3,5], and a clear answer will not be formally available until results of the on-going international trial START are available. At present, only observational studies have shown an individual benefit in terms of reduced morbidity when ART is initiated, independently of CD4 count, in the following situations: plasma viral load (VL) >5 log copies/mL, age >50 years, HIV/HBV or HIV/HCV co-infection, active comorbidities (tumour, nephropathy, neurological and cardiovascular disease).

Early treatment initiation is associated with better preservation of the immune system, which includes maintenance or restoration of a CD4 count >500/mm^3^ and of a CD4/CD8 ratio >1; preservation of central memory CD4 T-cells, of the diversity of the T-cell repertoire and notably of the HIV-specific T-cell response; and reduced HIV reservoirs and lymphoid tissue fibrosis [6–8]. Early treatment initiation also reduces chronic systemic inflammation resulting from uncontrolled HIV infection, which is known to have deleterious cardiovascular, metabolic, bone and neurologic effects, certain markers of which (in particular, soluble CD14) have been independently associated with mortality [9].

#### Reduction of HIV transmission risk

ART is an effective way of reducing HIV transmission, as shown in the trial HPTN052, with a 96% reduction in the risk of HIV transmission to the uninfected partner (95% CI: 73–99%) [10].

Reduction in the risk of sexual transmission of HIV is therefore a major goal of ART, both from an individual and a public health standpoint.

#### Potential drawbacks of early initiation of ART

The early initiation of ART should be discussed in light of its potential drawbacks: short-term adverse drug reactions, possible toxicity related to prolonged exposure to certain antiretrovirals, impact on patients’ quality of life, and treatment cost. However, these drawbacks have been constantly minimized over the recent years. It is also necessary to take into account the risk of suboptimal adherence to early ART, and hence the risk of emergence of resistant viruses, which would compromise future treatment options.

#### In conclusion

- ART should be initiated in any person living with HIV, whatever his/her CD4 T-cell count, even when >500/mm^3^.
- The level of evidence of the individual benefit of ART in terms of mortality or progression to AIDS differs according to CD4 count: high when the CD4 count is <500/mm^3^, moderate when the CD4 count is >500/mm^3^ (see Table 1).
- The early initiation of ART, whatever the CD4 count, is associated with other benefits: clinical (reduction of comorbidities associated with HIV infection), immunological (**BII**) and reduction of HIV transmission risk (**AI**).
- When the CD4 count is >500/mm^3^ and stable, treatment can be postponed if the patient does not immediately adhere to the treatment plan. In such a case, the physician, in conjunction with a patient education team and/or support group, should make every effort to prepare the patient for later implementation of treatment.

**Table 1:** Initiation of antiretroviral therapy in asymptomatic adults

ART should be initiated in any person living with HIV, irrespective of his/her CD4 count **(AII)**. The level of evidence of this recommendation depends on the patient’s situation at the start of treatment: • CD4 <350/mm^3^: **AI** • CD4 between 350 and 500/mm^3^: **AII** • CD4 >500/mm^3^: **BIII** • Primary infection: **BII**

Effective ART prevents HIV transmission from a person living with HIV person to his/her sexual partners. This information should be delivered patients living with HIV, and ART can be started with the aim of preventing sexual transmission of HIV (**AI** for transmission in a heterosexual couple, **BIII** for other situations).

### Patients diagnosed with primary infection

Recent data prompt recommendation of immediate treatment initiation during primary infection, whether or not it is symptomatic and regardless of CD4 count and VL (**BII**). It has been shown that ART is more likely to preserve immune functions and to decrease HIV reservoir when started at the time of primary infection rather than at the stage of chronic infection [7]. In addition, ART initiated early after infection is expected to significantly decrease a patient’s infectiousness, which is high during and early after primary infection. Outside research protocols, treatment initiated during the primary infection should not be stopped.

### Patients with a low VL (<1000 copies/mL)

Less than 1% of people show spontaneous and prolonged control of viral replication (HIV controllers). Usually, this virologic control is accompanied by prolonged maintenance of a high CD4 count (long-term non-progressors). Before considering any postponement of initiation of ART, it is necessary to ensure that the CD4 count is steadily above 500/mm^3^.

### Highly immunosuppressed patients (CD4 count <200/mm^3^) with no identified opportunistic infection

ART should be started quickly. In patients with a CD4 count <200/mm^3^, the clinical prognosis is better if the treatment is initiated at a higher CD4 count (**AI**) [11,12]. Screening for latent opportunistic infection is recommended to limit the risk of immune restoration inflammatory syndrome after the initiation of ART (**AI**). Prophylaxis of opportunistic infections should be undertaken.

### Patients with an opportunistic infection

In patients with an HIV-related complication (cognitive disorders, HIV encephalopathy) or an infection with no specific treatment (progressive multifocal leukoencephalopathy, cryptosporidiosis, microsporidiosis …), ART should be initiated without delay to restore specific immunity against the causal agent.

When the opportunistic infection may be cured with a specific treatment, the situation differs depending on the nature of the opportunistic infection. In infections by *Pneumocystis jirovecii*, *Toxoplasma gondii*, cytomegalovirus and other herpes viruses, ART should be started within two weeks of the start of treatment for the opportunistic infection [13], after making sure that the specific anti-infective treatment is well-tolerated (**AI**). Delay in initiating antiretrovirals exposes the patient to a high risk of another opportunistic infection, especially if the CD4 count is <50/mm^3^.

In tuberculosis with no meningeal involvement, the timing of initiation of ART depends on the degree of immune suppression. The benefit of early treatment, initiated within two weeks of the start of the antituberculosis treatment, is crucial when the CD4 count is <50/mm^3^, even if the risk of immune restoration inflammatory syndrome is greater in such patients [14] (**AI**). When the immune deficiency is less severe (CD4 count >50/mm^3^), the benefit of early treatment is not as clear and treatment can be postponed by two to four weeks (**AI**) [15,16].

In meningeal tuberculosis [17] and cryptococcal meningitis [18,19], the recommended interval between treatment of the opportunistic infection and ART is at least four weeks, provided the clinical course is good and cultures become negative for Cryptococcus (**AI**), with the objective to reduce excess immediate mortality by lowering the risk of immune restoration inflammatory syndrome (**AI**).

### What to start?

Table 2 summarizes the laboratory tests that should be performed before initiating the first session of ART.

**Table 2:** Laboratory tests performed prior to treatment of an adult living HIV

HIV serology: ELISA on two different samples with confirmation by Western blot of HIV1 (HIV2 if epidemiological context relevant)
CD4/CD8 T-cell count
Plasma HIV-RNA (viral load)
Genotypic testing for HIV drug resistance (reverse transcriptase, protease) and determination of the HIV-1 subtype (integrase resistance testing and testing of HIV tropism are not recommended at this stage)
HLA-B*5701 screening
Blood cell count with platelet count
ALT/AST, γGT, alkaline phosphatases, total and conjugated bilirubin
Blood creatinine and estimation of glomerular filtration rate (MDRD or CKD-EPI equation)
Fasting blood glucose
Blood phosphate
Fasting lipid profile: total cholesterol, triglycerides, LDL and HDL
Testing for proteinuria (urine dipstick) or determination of the protein/creatinine ratio
Markers of viral hepatitis B: HBs antigen, anti-HBs and anti-HBc antibodies
Serological testing for viral hepatitis C
Serological testing for viral hepatitis A (IgG)
Serological testing for syphilis (*Treponema pallidum* hemagglutination assay, Venereal Disease Research Laboratory test)
Serological testing for toxoplasmosis
CMV serology testing
IFN-gamma release assay (QuantiFERON or T-SPOT.TB) for detection of latent tuberculosis
If CD4 T-cell count <200/mm^3^ or person from an area where tuberculosis is endemic: chest X-ray
If CD4 T-cell count <100/mm^3^: cryptococcal antigen assay, blood CMV PCR test, and fundus examination (if CMV serology positive)
In women who have not had a gynaecological examination for one year, examination with a cervical screening test is recommended.
In men who have sex with men and in people living with HIV who have a history of human papillomavirus lesions, a proctological examination should be proposed to screen for precancerous lesions of the anus.

### Goals of the first antiretroviral treatment

The first session of ART should render the VL undetectable (<50 copies HIV-RNA/mL) in six months. During the first months of treatment, VL should be determined:

- at month 1, when the plasma VL (CV) should have decreased by at least 2 log copies/mL;
- at month 3, when the VL should be <400 copies/mL;
- at month 6, when the VL should be <50 copies/mL.

If these interim goals are not achieved, suboptimal adherence to treatment, possible drug interactions or under-dosing of antiretrovirals (notably by plasma assay of certain classes of drugs) should be searched for and corrected without delay.

In some patients, this goal is not achieved at these times and the VL only becomes undetectable after more than six months of treatment. This is seen notably when the initial VL is >5 log copies/mL or the CD4 count is <200/mm^3^. In these patients, if the VL is<200 copies/mL at six months and is decreasing regularly, it is possible to reach undetectability during close monitoring for four to six additional months without specific intervention.

### Considerations for the choice of the first antiretroviral therapy

In 2013, a first-line triple-drug therapy is a combination of two nucleoside reverse transcriptase inhibitors (NRTI) with a third drug. There are numerous options that have been validated in terms of immunological and virological efficacy.

The choice of the first treatment should be individualized with the patient, who should participate in this choice, with the aim of maximizing adherence. The first treatment is chosen in light of:

- the expected safety of the treatment,
- treatment convenience in view of the patient’s living conditions and lifestyle,
- anticipated drug interactions with other possible concomitant treatments,
- the patient’s comorbidities, in particular cardiovascular, renal, hepatic, addictive behaviour and psychiatric disorders, co-infection with tuberculosis,
- the results of the pre-treatment genotypic resistance test,
- the effects of failure on later treatment options,
- the results of screening for the HLA-B*5701 allele,
- finally, but importantly, the treatment cost (Table 3).

**Table 3:** Cost of antiretroviral drugs available in France in 2013*

Antiretroviral drug (Branded formulation – manufacturers)	Usual daily dosing (Adult)	Monthly cost (€)
**Nucleoside reverse transcriptase inhibitors**		
Abacavir (Ziagen^®^ – ViiV Healthcare)	300 mg×2 or 600 mg×1	286
Emtricitabine (Emtriva^®^ – Gilead Sciences)	200 mg×1	163
Didanosine (Videx^®^ – Bristol Myers Squibb)	≥60 kg: 400 mg×1	218
	<60 kg: 250 mg×1	135
Lamivudine (Epivir^®^ – ViiV Healthcare)	150 mg×2	181
Zidovudine (Retrovir^®^ – ViiV Healthcare)	300 mg×2	238
Nucleotide reverse transcriptase inhibitors		
Tenofovir (Viread^®^ – Gilead Sciences)	245 mg×1	366
**Non-nucleoside reverse transcriptase inhibitors**		
Efavirenz (Sustiva^®^ – Bristol Myers Squibb)	600 mg×1	315
Etravirine (Intelence^®^ – Janssen)	200 mg×2	505
Nevirapine (Viramune^®^ – Boehringer Ingelheim)	200 mg×1 for 14 days	56 (14 days)
	then 200 mg×2	226
	or 400 mg LR×1/j	280
Rilpivirine (Edurant^®^ – Janssen)	25 mg×1	270
**Protease inhibitors**		
Atazanavir/ritonavir (Reyataz^®^/Norvir^®^) – (Bristol Myers Squibb/Abbott)	300/100 mg×1 or	455/27
	400 mg×1 (non-ritonavir-boosted)	455
Darunavir/ritonavir(Prezista^®^/Norvir^®^)– (Janssen/Abbott)	ARV naive: 800 mg/100×1	490/27
	ARV experienced: 600 mg/100×2	735/54
Fosamprenavir/ritonavir (Telzir^®^/Norvir^®^) – (ViiV Healthcare/Abbott)	700/100 mg×2	377/54
Lopinavir/ritonavir (Kaletra^®^ – Abbott)	400/100 mg×2	476
Saquinavir/ritonavir (Invirase^®^/Norvir^®^) – (Roche/Abbott)	1000/100 mg×2	399/54
Tipranavir/ritonavir (Aptivus^®^/Norvir^®^) – (Boehringer–Ingelheim/Abbott)	500/200 mg×2	810/108
**Integrase inhibitor**		
Raltegravir (Isentress^®^ – Merck)	400 mg×2	700
**CCR5-antagonist**		
Maraviroc (Celsentri^®^ – ViiV Healthcare)	50 to 600 mg×2	729 to 1684
**Fusion inhibitor**		
Enfuvirtide (Fuzeon^®^ – Roche)	90 mg×2 (subcutaneous injections)	1684
**Fixed-dose co-formulations**		
Abacavir+lamivudine (Kivexa^®^ – ViiV Healthcare)	600 mg+300 mg	412
Tenofovir+emtricitabine (Truvada^®^ – Gilead Sciences)	245 mg+200 mg	520
Tenofovir+emtricitabine+efavirenz (Atripla^®^ – Bristol Myers Squibb)	245 mg+200 mg+600 mg	746
Tenofovir+emtricitabine+rilpivirine (Eviplera^®^ – Gilead Sciences)	245 mg+200 mg+25 mg	756

* http://medicprix.sante.gouv.fr/medicprix/rechercheSpecialite.do?parameter=rechercheSpecialite (Accessed Aug 6, 2013).

### Regimens recommended as first antiretroviral therapy

The following considerations were taken into account by the expert group in defining the preferred treatment options:

- efficacy and safety of treatment regimens evaluated in well-conducted randomized trials,
- simplicity of administration (mainly one vs. two intakes)
- treatment cost (mean annual cost of ART is estimated in France to be around €10,800, i.e. US$ 13,500).

Table 4 summarizes the preferred combinations for the initiation of a first session of ART.

The case for the choice of the recommended regimens is presented afterwards.

**Table 4:** Options recommended for the initiation of a first session of ART

Preferred choices – no order of preference			

2 NRTIs	NNRTIs		Comments
Tenofovir DF/emtricitabine 1 tab/day	Efavirenz 600 mg×1	**AI**	Available as STR Renal monitoring. Precautions if creatinine clearance<80 mL/min. Efavirenz not to be prescribed to women who are pregnant or are likely to become so
Tenofovir DF/emtricitabine 1 tab/day	Rilpivirine 25 mg×1	**AI**	Available as STR Renal monitoring. Precautions if creatinine clearance<80 mL/min. Only if VL <5 log copies/mL Precautions if CD4 count <200/mm^3^ Should be taken with a meal
Abacavir/lamivudine 1 tab/day	Efavirenz 600 mg×1	**AI**	Efavirenz not to be prescribed to women who are pregnant or are likely to become so Only if VL <5 log copies/mL Only if HLA-B*5701 negative

**2 NRTIs**	**Ritonavir-boosted protease inhibitor**		

Tenofovir DF/emtricitabine 1 tab/day	Atazanavir/r 300/100 mg×1	**AI**	Close renal monitoring. Precautions if creatinine clearance <80 mL/min.
Tenofovir DF/emtricitabine 1 tab/day	Darunavir/r 800/100 mg×1	**AI**	Close renal monitoring. Precautions if creatinine clearance <80 mL/min.
Abacavir/lamivudine 1 tab/day	Atazanavir/r 300/100 mg×1	**AI**	Only if VL <5 log copies/mL Only if HLA-B*5701 negative

**Other possible choices – no order of preference**			

**2 NRTIs**	**NNRTIs**		**Comments**

Abacavir/lamivudine 1 tab/day	Rilpivirine 25 mg×1	**BII**	Only if VL <5 log copies/mL Only if HLA-B*5701 negative Precautions if CD4< 200/mm^3^ Should be taken with a meal
Tenofovir DF/emtricitabine 1 tab/day	Nevirapine 400 mg/day	**BI**	Renal monitoring. Precautions if creatinine clearance<80 mL/min. If CD4 <250/mm^3^ for women and <400/mm^3^ for men

**2 NRTIs**	**Ritonavir-boosted protease inhibitor**		

Tenofovir DF/emtricitabine 1 tab/day	Lopinavir/r 400/100 mg×2	**BI**	Close renal monitoring. Precautions if creatinine clearance <80 mL/min. Precautions if high cardiovascular risk
Abacavir/lamivudine 1 tab/day	Lopinavir/r 400/100 mg×2	**BI**	Precautions if high cardiovascular risk Only if HLA-B*5701 negative
Abacavir/lamivudine 1 tab/day	Darunavir/r 800/100 mg×1	**BIII**	Only if HLA-B*5701 negative

2 **NRTIs**	**Integrase inhibitor**		

Tenofovir DF/emtricitabine 1 tab/day	Raltegravir 400 mg×2	**BI**	Renal monitoring. Precautions if creatinine clearance<80 mL/min. Raltegravir rarely a source of interactions Two daily doses High cost of raltegravir
Abacavir/lamivudine 1 tab/day	Raltegravir 400 mg×2	**BI**	Raltegravir rarely a source of interactions Two daily doses High cost of raltegravir Only if HLA-B*5701 negative

NRTIs: nucleoside reverse transcriptase inhibitors; NNRTIs: non-nucleoside reverse transcriptase inhibitors; STR: single-tablet regimen once daily.

### Choice of the two nucleoside/nucleotide reverse transcriptase inhibitors of the triple-drug therapy

Two fixed-dose combinations of NRTIs are recommended because of their efficacy, safety and simplicity of use (one tablet a day): tenofovir disoproxil fumarate/emtricitabine and abacavir/lamivudine.

#### Tenofovir DF/emtricitabine

This combination is more effective virologically and immunologically and better tolerated than zidovudine/lamivudine in combination with efavirenz [20,21]. It is the combination most often used in development trials of new third drugs (raltegravir, rilpivirine, elvitegravir/cobicistat). The nephrotoxicity of tenofovir DF may manifest as decreased glomerular filtration rate and/or proximal tubulopathy with proteinuria, glycosuria and hypophosphatemia. The risk of nephrotoxicity is greater in patients with advanced HIV infection, pre-existing nephropathy (HIV-associated nephropathy, in particular) and when treatment includes a PI/r or cobicistat, both of which increase plasma concentrations of tenofovir DF [22,23]. Creatinine clearance should be calculated in all patients before initiation of tenofovir DF treatment and renal function should be tested regularly (creatinine clearance and phosphoremia).

As emtricitabine and tenofovir are active against HBV, the patient’s HBV serostatus should be tested before prescribing this combination.

In people co-infected by HBV (Ag HBs positive or isolated anti-HBc antibody), unless there is a contraindication, the combination tenofovir DF/emtricitabine is recommended because of its anti-HBV activity.

#### Abacavir/lamivudine

This combination also has the advantage of ease of use and safety. The risk of abacavir hypersensitivity syndrome (approximate incidence 5%) is the main drawback of this combination, but this risk can be virtually eliminated by screening for the HLA-B*5701 allele and contraindicating abacavir in people positive for this allele [24]. Its efficacy and safety have been confirmed in several trials, in combination with efavirenz, atazanavir/r or lopinavir/r.

In the ACTG5202 trial, in which screening for the HLA-B*5701 allele was not done before treatment initiation, abacavir/lamivudine proved less effective than tenofovir DF/emtricitabine in people with a VL >5 log copies/mL, whether in combination with atazanavir/r or efavirenz [25] In people with a VL<5 log copies/mL, there was no difference between abacavir/lamivudine and tenofovir DF/emtricitabine in terms of virological efficacy, whether in combination with atazanavir/r or efavirenz. Abacavir/lamivudine was also virologically less effective than tenofovir DF/emtricitabine in the ASSERT trial [26]. In contrast, in the HEAT trial, the non-inferiority of abacavir/lamivudine compared to tenofovir DF/emtricitabine, in combination with lopinavir/r, was demonstrated in terms of virological efficacy, whatever the VL at inclusion [27].

Several studies have explored the link between abacavir and myocardial infarction, with inconsistent results. The pathophysiological mechanism by which abacavir would increase the risk of myocardial infarction remains unknown.

In the light of currently available data, it seems reasonable to limit the use of abacavir/lamivudine in treatment initiation to patients with a VL <100,000 copies/mL where abacavir/lamivudine is an alternative to tenofovir DF/emtricitabine, in particular in patients at renal risk.

#### Zidovudine/lamivudine

Studied in many trials, zidovudine/lamivudine is the combination with which we have the greatest experience. Its efficacy has been proven in several triple-drug therapies. It is available in the form of a fixed-dose combination at the dose of one tablet twice a day. The most frequent adverse drug reactions are those of zidovudine (gastrointestinal intolerance, headache, anaemia, myopathy and mitochondrial toxicity). Mitochondrial toxicity manifests clinically as a greater frequency of lipoatrophy compared with the combination tenofovir DF/emtricitabine [20]. Zidovudine/lamivudine should no longer be used within a first-line regimen, except in special cases (pregnant women, HIV encephalitis, patients who would both be positive for HLA-B*5701 and have chronic renal failure).

#### Other combinations of two NRTIs

These are less favourable in terms of efficacy and safety and should no longer be used in first-line regimens. The same is true of the triple-drug therapy zidovudine/lamivudine/abacavir.

#### In conclusion

- The combinations of tenofovir DF/emtricitabine and abacavir/lamivudine should be preferred in a first triple-drug therapy.
- The combination tenofovir DF/emtricitabine should be preferred if VL is ≥5 log copies/mL.
- When VL is <5 log copies/mL, the choice between abacavir/lamivudine and tenofovir DF/emtricitabine can be made on a case-by-case basis, taking into account co-infection with HBV and renal function.

In people co-infected by HBV (Ag HBs positive or anti-HBc antibody isolated) and unless there is a contraindication, the combination tenofovir DF/emtricitabine is recommended because of its anti-HBV activity.

The combination tenofovir DF/emtricitabine should be used with caution when creatinine clearance is <80 mL/min or if there is a risk of renal failure, notably when combined with another nephrotoxic drug. It should be avoided, special cases apart, if creatinine clearance is <60 mL/min and it is contraindicated if creatinine clearance is <30 mL/min.

- The combination of abacavir/lamivudine should only be used in patients who do not carry the HLA-B*5701 allele.

### The choice of the third drug

#### Triple-drug therapy with protease inhibitor as the third drug

The use of a protease inhibitor can only be considered if it is boosted by the addition of a low dose (100 to 200 mg/day) of ritonavir (PI/r). Because HIV has a higher genetic barrier to protease inhibitors than to NNRTIs, early resistance to protease inhibitors is rare when plasma concentrations of PI are insufficient (because of suboptimal adherence).

Atazanavir/r (300/100 mg once a day) demonstrated non-inferiority to lopinavir/r in combination with tenofovir DF/emtricitabine. The plasma lipid profile was slightly better for atazanavir/r [28,29].

Darunavir/r (800/100 mg once a day) was globally not inferior to lopinavir and even superior to lopinavir in patients with an initial VL >5 log copies/mL. Clinical tolerance, notably gastrointestinal, and plasma lipid profile are better [28,30].

Lopinavir is co-formulated with ritonavir (200/50 mg or 100/25 mg per tablet). The standard dose in adults is 400/100 mg twice a day. A once daily regimen (800/200 mg) has the same immunological and virological efficacy as the conventional regimen, at the expense of a poorer tolerability (diarrhoea) [30].

#### In conclusion

If a ritonavir-boosted protease inhibitor (PI/r) is chosen as third drug, atazanavir/r or darunavir/r should be preferred.

#### Triple-drug therapy with an NNRTI as the third drug

Three NNRTIs can be used in first-line regimens: efavirenz, nevirapine and rilpivirine. This type of regimen has two main drawbacks:

- the rate of primary resistance to NNRTIs (7.1% in 2012 in the French Primo cohort), which means that the result of the genotypic resistance test has to be available before starting such a treatment regimen;
- the low genetic barrier of HIV to NNRTIs, which leads to the risk of rapid selection of viruses resistant to all first-generation NNRTIs (efavirenz, nevirapine) and to NRTIs (mainly lamivudine and emtricitabine) included in the treatment regimen. Etravirine usually remains active against viruses with resistance mutations to first-generation NNRTIs. However, the mutations selected by exposure to rilpivirine may confer cross-resistance to etravirine (mutation at codon 138 of the reverse transcriptase).

##### Efavirenz

The ACTG A5142 trial compared a triple-drug therapy with efavirenz with a triple-drug therapy with lopinavir/r: the virologic response was greater in the efavirenz arm, but in the lopinavir/r arm the immune response was better, and there was less resistance in the case of failure and less lipodystrophy [31].

The ACTG 5202 trial compared in a double-blind design efavirenz and atazanavir/r in combination with either abacavir/lamivudine or tenofovir DF/emtricitabine [25,32]. Similar virological efficacy was demonstrated between atazanavir/r and efavirenz. In combination with abacavir/lamivudine, atazanavir/r was better tolerated than efavirenz. In combination with tenofovir DF/emtricitabine, the increase in CD4 count at weeks 48 and 96 was greater with atazanavir/r than with efavirenz.

Efavirenz is frequently associated with neurosensory adverse reactions, but these are usually transient at the start of treatment, mood disorders may develop later. These neurological adverse drug reactions may be more frequent in people from Africa or Asia, who more often carry a non-functional variant of CYP2B6 (CYP2B6516TT), resulting in higher plasma concentrations [33]. Efavirenz is contraindicated during the first trimester of pregnancy.

The ENCORE 1 trial recently showed a similar virological efficacy for efavirenz administered at the dosage of 400 or 600 mg/day (in combination with tenofovir DF/emtricitabine) [R Puls *et al*., IAS 2013, Kuala Lumpur, Malaysia, abstract WELBB01].

##### Nevirapine

The open label ARTEN trial compared nevirapine with atazanavir/r in combination with tenofovir DF/emtricitabine, with the usual restrictions for use of nevirapine in treatment-naive patients (CD4 count <250/mm^3^ in women and <400/mm^3^ in men, see below). In terms of virological efficacy, the non-inferiority of nevirapine was demonstrated, whether administered twice or once daily. The safety of nevirapine was slightly better in terms of plasma lipid profile [34,35].

In treatment-naive patients, to reduce the risk of hypersensitivity, Nevirapine should not be used if the CD4 count is above 400/mm^3^ in men and 250/mm^3^ in women. Start with a half-dose for the first two weeks, that is, 200 mg once a day and measure transaminases every two weeks for the first two months of treatment, at the third month and regularly afterwards. All of these restrictions mean that nevirapine is difficult to use within a first-line regimen [36].

##### Rilpivirine

Rilpivirine at the dose of 25 mg once a day has been studied in two randomized, double-blind trials versus efavirenz in combination with tenofovir DF/emtricitabine or abacavir/lamivudine [37,38]. In the overall analysis of these two trials, the non-inferiority of rilpivirine in terms of virological efficacy was demonstrated at 96 weeks (76 and 77% of patients had a VL <50 copies at 96 weeks on rilpivirine and efavirenz, respectively) [39]. However, the rate of virologic suppression was lower when VL was >5 log copies/mL and CD4 count was below 200/mm^3^. In case of virologic failure, viruses isolated at failure more often presented resistance mutations to other NNRTIs and to emtricitabine/lamivudine and tenofovir after failure with rilpivirine than after failure with efavirenz. In contrast, discontinuation because of adverse drug reactions and worsening of plasma lipid profile were less frequent with rilpivirine than with efavirenz.

Rilpivirine is co-formulated in a fixed-dose combination (tenofovir DF/emtricitabine/rilpivirine), and one tablet per day should be taken at mealtime. The concomitant use of proton pump inhibitors is contraindicated, and H2 antihistamines should be limited because of a risk of decreased plasma rilpivirine concentration. This combination was evaluated in a randomized open trial (STAR) versus tenofovir DF/emtricitabine/efavirenz and given as a STR. Non-inferiority was demonstrated in all patients studied, with superiority of tenofovir DF/emtricitabine/rilpivirine when VL was below 5 log copies/mL at inclusion. The rate of virologic failure was identical in the two treatment arms, but the rate of resistance mutations on treatment failure was higher in the tenofovir DF/emtricitabine/rilpivirine arm than in the tenofovir DF/emtricitabine/efavirenz arm when VL at inclusion was >5 log copies/mL. Clinical safety and laboratory safety data were better with tenofovir DF/emtricitabine/rilpivirine than with tenofovir DF/emtricitabine/efavirenz [40].

##### In conclusion

If an NNRTI is chosen as a third drug for the initiation of the first session of ART, efavirenz should be preferred, or rilpivirine if the VL before treatment initiation is <5 log copies/mL. Each is used in a once daily fixed-dose combination tablet.

#### Triple-drug therapy with an integrase inhibitor as the third drug

Raltegravir: Raltegravir is the first in the integrase inhibitor class with a dosage of twice a day. At the dose of 400 mg twice a day, raltegravir was compared with efavirenz in combination with tenofovir DF/emtricitabine in 566 treatment-naive patients in STARTMRK, a randomized, double-blind trial. The non-inferiority of raltegravir was demonstrated in terms of virological efficacy. Safety was significantly better with raltegravir than with efavirenz [41]. VL decreased faster with raltegravir than with efavirenz [41,42]. Raltegravir was not compared with a PI/r and was only evaluated with abacavir/lamivudine in a non-comparative pilot trial in a small number of patients [43]. The risk of selection of resistant variants in the event of virologic failure was greater and occurred faster than with a treatment including a PI/r. The safety of raltegravir is generally good, but rare serious adverse drug reactions have been reported: rash, hypersensitivity syndrome, myositis and rhabdomyolysis, depression. Finally, raltegravir is more expensive than other currently available third drugs.

In conclusion, although the efficacy and safety of tenofovir DF/emtricitabine/raltegravir have been demonstrated in a well-conducted randomized trial, this combination is not preferred because of its high cost (in 2013) and its twice-daily dosing.

Elvitegravir/cobicistat and Dolutegravir: Although randomized trials have demonstrated the efficacy and safety of elvitegravir/cobicistat in fixed-dose combination with tenofovir DF/emtricitabine [44,45] and of dolutegravir in combination with tenofovir DF/emtricitabine or abacavir/lamivudine [46,47], the expert group made no recommendations regarding these new options, which were not yet available and for which pricing details were not provided [45].

## Specific situations for the choice of the first session of ART

### Women

Before initiating ART in a woman, she should first be asked whether she plans to become pregnant:

- if she does, and does not use contraception, a regimen without NNRTIs should be preferred, because of the neurological risk associated with exposure of the embryo to efavirenz, the risk of nevirapine hypersensitivity and hepatotoxicity, and the lack of experience with rilpivirine in early pregnancy.
- if she is not planning a pregnancy and is using a contraceptive method or is menopausal, it is necessary to check potential interactions with oestrogen–progesterone oral contraceptives, the efficacy of which can be reduced by PI/r and NNRTIs, or the exposure increased with a protease inhibitor without ritonavir (atazanavir), with greater toxicity (thromboembolic risk).

### Primary infection

The choice of treatment at the time of primary infection will be affected by the need to initiate treatment quickly and by the epidemiology of resistance. In general, no information is available on screening for the HLA-B*5701 allele or on genotypic resistance testing. The choice of a triple-drug therapy will therefore favour the combination tenofovir DF/emtricitabine, in the absence of kidney disease, because the VL is frequently high, and of a PI/r, as primary resistance to NNRTIs is about 7% in France and the treatment should be started quickly at a time when the results of resistance genotyping are not known.

### Severe immune suppression (CD4 count <200/mm^3^) without identified opportunistic infection

Here, the choice is based on the same options as those for asymptomatic patients, taking into account the fact that a low CD4 count is generally associated with a high VL. The question then is whether to intensify the treatment with a fourth drug to reduce viral replication more rapidly, with the aim of faster immune restoration. Trials that have tested four-drug therapies combining two NRTIs, a protease inhibitor and an NNRTI have shown no benefit over triple-drug therapy, while more frequent adverse drug reactions were observed [48].

### Diagnosis and treatment of a major opportunistic infection

The choice is based on the same options as those for asymptomatic patients, considering the fact that a low CD4 count is generally associated with a high VL on the same options, taking into account CD4 count and VL, immediate adverse drug reactions and possible interactions between the treatment of the opportunistic infection and the antiviral treatment.

In the case of tuberculosis treated with rifampicin, the options for the third drug are efavirenz at standard dose (600 mg/day) or raltegravir at standard dose (400 mg twice a day). The prescription of a PI/r implies that rifampicin has to be replaced by low-dose rifabutin (150 mg every two days). The prescription of other antiretrovirals (rilpivirine, etravirine or elvitegravir) is not recommended because of a strong decrease in antiretroviral concentrations and the attendant risk of virologic failure, whether in combination with rifampicin or rifabutin.

In opportunistic infections other than tuberculosis, the third drug is chosen in the same way as for highly immunosuppressed patients. Because of the risk of interactions between certain anti-infectious treatments and antiretrovirals, antiviral drug concentrations should be determined with a view to adjusting the dose.

### Injection or substitution drug users

The risk of pharmacological interactions between antiretrovirals and injectable or substitution drugs (methadone, buprenorphine) should be taken into account when choosing antiretrovirals. Enzyme inducers (efavirenz, nevirapine) generate the risk of under-dosing of morphine-like drugs, the dose of which, if used, should be increased. Conversely, enzyme inhibitors (ritonavir, cobicistat, protease inhibitors) generate the risk of over-dosing of certain intoxicants (notably ecstasy and *gamma*-hydroxybutyric acid [GHB] metabolized by cytochrome CYP2D6 and certain benzodiazepines metabolized by cytochrome CYP3A4) [49].

## Optimization of ART after virologic suppression

When a sustained virologic suppression has been achieved on ART (VL under the limit of quantification for more than 6 months), treatment optimization can be proposed. The aim is to improve quality of life, promote long-term adherence, and prevent virologic failure.

### Change of NRTI

Most current antiretroviral combinations include two NRTIs and a third drug. There is greater toxicity, notably mitochondrial (lipodystrophy, hepatic steatosis), with first-generation NRTIs, which should be replaced by more recent NRTIs, available as once daily single tablet fixed-dose combinations (tenofovir DF/emtricitabine or abacavir/lamivudine). Stavudine should no longer be used. Didanosine and zidovudine should also be replaced.

### Change of the third drug

When the third drug is a PI/r, it can be changed in the case of intolerance, or in prevention of long-term toxicity.

#### Change of PI/r

Darunavir/r and atazanavir/r are easier to use than older PIs/r (once a day, fewer tablets, lower dose of ritonavir) and have a better gastrointestinal safety. In the absence of previous failure of a treatment including a PI/r, it is easy to replace an old PI/r by darunavir/r or atazanavir/r.

The use of atazanavir without ritonavir can also be considered, at the dose of 400 mg/day. The aim is to improve the safety of the treatment by withdrawing ritonavir (hyperbilirubinemia, gastrointestinal disorders, hyperlipidemia). Virological efficacy has been maintained up to 144 weeks [50] and is associated with an improved lipid profile [50,51]. In practice, the full efficacy of the atazanavir plus NRTIs (prior resistance genotyping, history of failure on PI/r) should be checked. Therapeutic drug monitoring is recommended in the case of combination with tenofovir/DF, which can reduce atazanavir plasma concentration.

#### Replacement of the PI/r by an NNRTI

Several randomized trials have shown the efficacy of such a treatment strategy, with efavirenz or nevirapine. The replacement of PI/r by rilpivirine was evaluated in a randomized, open, non-inferiority trial in 476 patients with virologic suppression for over six months on a first- or second-line triple-drug therapy combining two NRTIs and a PI/r. Patients in the intervention arm received the fixed-dose combination rilpivirine/tenofovir DF/emtricitabine once daily; patients in the control arm continued the treatment they were taking at the time of enrolment. Non-inferiority was demonstrated. The rate of virologic failure was lower in the rilpivirine/tenofovir DF/emtricitabine arm (0.9%) than in the control arm. These strategies have the advantage of simplifying treatment, notably in terms of number of tablets, and of improving safety (gastrointestinal, lipid profile, and long-term cardiovascular safety for nevirapine). It is essential to check that the NRTI to be combined with NNRTIs is fully active (analysis of treatment history, testing for viral replication on NNRTI therapy, analysis or repeat analysis of available resistance genotypes). The initiation of nevirapine in a treatment switch in patients with virologic suppression does not call for the same precautions concerning CD4 count as on the introduction of a first treatment [52].

#### Replacement of the PI/r by raltegravir

Replacement of the PI/r by raltegravir also simplifies the on-going treatment regimen and improves safety (lipid profile), but it requires two doses a day and, as for NNRTIs, necessitates a check that there are no resistance mutations to the NRTIs taken by the patient, because of the risk of virologic rebound when the combined antiretrovirals are not fully active [53,54].

### Treatment regimens without NRTIs

#### PI/r monotherapy

Maintenance strategies using PI/r monotherapy have been investigated in numerous randomized or cohort studies, mainly for lopinavir/r and darunavir/r. A meta-analysis of 10 randomized trials (lopinavir/r: 7, darunavir/r: 2 and saquinavir/r: 1) including 1189 patients showed that PI/r monotherapy was less virologically effective than maintenance of the on-going triple-drug therapy [55]. However, the difference in efficacy was small and perhaps dependent on the choice of PI/r. Monotherapy did not increase the risk of resistance and the re-introduction of NRTIs led to virologic suppression in 93% of cases.

A first trial with atazanavir/r in maintenance monotherapy recorded five virologic failures after the inclusion of 15 patients, leading to discontinuation of the study [56,57]. An observational study in the FHDH cohort (529 patients on maintenance monotherapy with darunavir/r (148), lopinavir/r (312) or atazanavir/r (69)) confirmed a higher risk of virologic failure with atazanavir/r [56].

These studies have identified the factors associated with maintained virologic suppression on PI/r monotherapy: use of lopinavir/r or darunavir/r, absence of previous virologic failure on protease inhibitor, prolonged virologic suppression on triple-drug therapy, low HIV-DNA (<2.3 log/10^6^ PBMC) and good adherence to treatment.

This strategy can be considered on a case-by-case basis.

#### Combination of PI/r +raltegravir

As atazanavir inhibits UGT 1A1, the metabolic pathway of raltegravir, there is a positive interaction between these two antiretrovirals. A pilot study in 25 virologically supressed patients under an atazanavir-based regimen evaluated raltegravir 400 mg bid+atazanavir 300 mg bid for four weeks followed by raltegravir 800 mg+atazanavir/r 300/100 mg once daily for four weeks or vice versa. Plasma HIVRNA remained undetectable in all patients, but the plasma concentrations of the two antiretrovirals were highly variable: lower for raltegravir when given q.d. and for atazanavir when given without ritonavir [58]. No recommendations can be made about this combination until the results of on-going randomized trials become available.

#### Combination of PI/r+maraviroc

Pharmacokinetic evaluation of the combination maraviroc (150 or 300 mg once a day)+darunavir/r (800/100 mg once a day) reveals an interesting pharmacokinetic profile, notably at the maraviroc dosage of 300 mg/day, which is similar to that of maraviroc 300 mg twice a day combined with tenofovir/emtricitabine [59]. No recommendations can be made about this combination until the results of these randomized trials become available.

### Regimens with neither NRTI nor protease inhibitor

#### Combination of raltegravir+maraviroc

The ANRS ROCnRAL trial (Katlama C, 20th CROI, Atlanta, 2013, abstract 566) has evaluated the efficacy of the combination raltegravir (2×400 mg/day)+maraviroc (2×300 mg/day) in 44 patients with a VL <50 copies/mL (R5-tropic virus) on triple-drug therapy including two NRTIs, and presenting lipodystrophy. The study was interrupted after the occurrence of five virologic failures, with selection of resistance mutations to integrase in three cases, a change in tropism (from R5 to X4) in one case and two serious adverse drug reactions. Consequently, this combination cannot be recommended.

#### Combinations of integrase inhibitors+NNRTIs

The success of switching to the combinations of raltegravir+nevirapine and raltegravir+etravirine has only been evaluated in a small number of patients in non-comparative studies. No recommendations can be made about such combinations until results of randomized clinical trials are available.

## Management of virologic failure

The aim of ART in all situations (first-line, later line, including after multiple failures) should be to reduce VL to <50 copies/mL and keep it there.

### Definitions

*Non-response to treatment* is defined as a less than 2-log reduction in VL one month after the introduction of the first session of treatment, or a less than 1-log reduction one month after the introduction of a treatment of suboptimal efficacy prescribed when there is virologic failure with multiple resistance.

*Initial failure* is defined as the persistence of a detectable VL (confirmed >200 copies/mL and confirmed >50 copies/mL, respectively, at six and twelve months after treatment initiation). The higher the VL at treatment initiation, the longer it takes to reduce it to an undetectable level (<50 copies/mL).

*Virologic rebound* is defined as an increase in VL to >50 copies/mL after a period of virologic suppression, confirmed for two consecutive samples, and should be distinguished from simple blips (transient, low-level viremia in a single sample). This blip usually corresponds to an isolated replication accident, often following a period of less strict adherence or an intercurrent infectious episode. Blips have no consequences in terms of the risk of later virologic failure or CD4 count [60–62], and should not lead to therapeutic intervention, apart from improving treatment adherence if necessary or testing for a pharmacological problem (absorption, drug interaction).

Depending on the detection threshold of the technique used to determine VL, some patients have a *low but repeatedly quantifiable VL* (>20 copies/mL). The consequences and the management of such situations are described below in “Management of low viral replication (VL <200 copies/mL).”

### Evaluation of virologic failure

The consequences and the management of virologic failure differ according to the level of viral replication. In all cases of virologic failure, the following actions should be taken:

- confirm the virologic failure: any detectable VL should be checked quickly; a second detectable measurement confirms virologic failure and distinguishes it from a virologic blip,
- measure the CD4 count, check its nadir and evaluate the patient’s clinical condition,
- evaluate adherence, notably looking for factors associated with poorer observance: adverse drug reactions, psychological difficulties, neurocognitive disorders, addictions and social vulnerability…
- perform a pharmacological evaluation: suitability of the doses and observance of timing of doses, notably when taken at mealtimes; testing for drug interactions; assays of residual plasma antiretroviral concentrations.
- collect the patient’s treatment history and analyze previous virologic failures and tolerability of previous drug regimens. In the case of a history of virologic failure on a treatment, including an NNRTI, an integrase inhibitor or lamivudine/emtricitabine, there may be resistance to these antiretrovirals, even if it has not been evidenced by a genotypic test.
- review resistance by collecting the results of previous genotypic resistance tests and by performing yet another test on plasma HIV RNA, always including analysis of the genes of reverse transcriptase and protease. Plus, depending on the case, analyze the genes of the integrase or of the viral envelope in the gp41 region in cases of prior exposure to integrase inhibitors or to fusion inhibitors, respectively. A tropism test can also be done to look for CXCR4-tropic virus, which would contraindicate the use of CCR5 inhibitors. Genotypic resistance tests are ideally done during the treatment that has resulted in virologic failure and are interpreted according to the latest recommendations of the ANRS group AC11 (www.hivfrenchresistance.org). If VL is <1000 copies/mL, problems with HIV-RNA amplification are likely and a larger volume of plasma can be concentrated to increase test sensitivity. In the case of failed amplification of plasma HIV-RNA, after consulting with a virologist, a genotypic resistance test may be performed on HIV-DNA in peripheral blood cells.

### Management of low viral replication (VL <200 copies/mL)

The consequences of residual viral replication (VL between 50 and 200 copies/mL) are not as clearly established as when replication leads to a VL >200 copies/mL. The same is true for replication below the lowest detection thresholds of current routinely used techniques (i.e. 20 copies/mL). If the patient receives a treatment including antiretrovirals with a low genetic barrier (lamivudine or emtricitabine, NNRTIs, integrase inhibitors), the risk of selecting additional resistance mutations increases with the level of viral replication and its duration [63,64]. This risk is lower if the patient receives PI/r treatment but may nonetheless exist if antiretrovirals combined with the PI/r are not fully active [65].

The priority is to eliminate known causes of virologic failure. If there are adherence problems not related to adverse drug reactions, a change of treatment is not generally the most appropriate response. Simplification of the treatment (number of doses, number of tablets) may improve adherence. If viral replication persists, particularly if VL increases and approaches 200 copies/mL, and if the patient is receiving NNRTIs or integrase inhibitors, a change of treatment should be considered, so as to prevent the selection of new mutations. In this situation, a resistance genotype test is most often not possible for a VL <200 copies/mL, because of failure of amplification (55% failure of amplification when the VL is between 51 and 500 copies/mL). The preferred choice will be a treatment including a PI/r.

### Management of virologic failure (VL >200 copies/mL)

Persistence of viral replication (VL >200 copies/mL) under antiretroviral selective pressure creates the risk of selection and accumulation of resistance mutations, plus immunological deterioration, which may lead to clinical progression and increased HIV transmission risk.

Rapid intervention is necessary whatever the CD4 count. The priority is to eliminate identified causes of virologic failure, such as a problem of adherence or a pharmacological cause. If the genotypic resistance test reveals new resistance mutations, rapid intervention is needed to avoid their accumulation, in particular with NNRTIs or integrase inhibitors, because of the risk of decreased efficacy of second-generation drugs of these classes [66]. The choice of the new treatment is ideally discussed at a multidisciplinary meeting of clinicians, a virologist and a pharmacologist. Treatment interruption is not recommended.

With currently available antiretrovirals, the aim of controlling viral replication (VL <50 copies/mL) can be achieved in most cases, including in patients with a long history of ART and the presence of more than one class of resistance mutations. Whenever possible, combinations evaluated in clinical trials should be preferred.

The optimal situation is when a treatment regimen containing three active drugs can be devised, based on treatment history and all the genotype data.

An antiretroviral can be considered active if it belongs to a class:

- not used previously;
- already used but for which the current resistance genotype and all the genotype data suggest it is active.

The new treatment will preferably combine an active PI/r (essentially darunavir/r in two daily 600 mg doses; more rarely tipranavir/r; a combination of two protease inhibitors is not recommended), with two other active antiretrovirals chosen from among:

- etravirine (which frequently remains active even when there is resistance to efavirenz and/or nevirapine, whereas there is cross-resistance with rilpivirine);
- raltegravir;
- dolutegravir 50 mg twice a day for patients with a virus presenting resistance mutations to raltegravir [67]. The SAILING trial has recently shown that dolutegravir (50 mg once a day) is virologically superior to raltegravir (400 mg×2/day) in patients experiencing treatment failure and in patients naive to treatment with integrase inhibitors [68].
- maraviroc, provided a tropism test done at the time of failure does not reveal a virus using the CXCR4 co-receptor
- enfuvirtide (though its injectable form limits prolonged use)
- one or more NRTIs. In the case of multiple resistance to NRTIs (≥3 thymidine analogue mutations+184V mutation), there may be residual activity of abacavir and tenofovir. All clinical trials performed with new antiretrovirals during the period 2002–2009 included optimized treatment with NRTIs, suggesting their possible contribution to the observed efficacy. In view of their good safety and ease of use, the maintenance of lamivudine or emtricitabine in the presence of the mutation M184V can be envisaged to maintain lower viral replication capacity.

In the TRIO trial, the combination of darunavir/r (600/100 mg twice a day), etravirine and raltegravir, with or without NRTIs and enfuvirtide, reduced VL to <50 copies/mL in 86% of patients by the 48th week [69].

When VL is <4 log copies/mL, a partial change including at least two active drugs, one of which is a PI/r, can be sufficient [70,71].

Multiple resistance is less and less frequent in recent years in France where <1% of patients have viruses resistant to all NRTIs and protease inhibitors [66]. In the currently exceptional situation where just one antiretroviral remains active, it is preferable to avoid functional monotherapy, which would lead to rapid selection of new resistance mutations. The possibility of using drugs in development for these patients, in the framework of clinical trials and/or temporary authorization for use, should be explored.

If genotypic resistance testing reveals resistance to all available drugs, the on-going treatment should not be changed pending the possibility of offering active multidrug therapy. It is sometimes useful to increase the doses of PIs/r, while monitoring plasma concentrations, to obtain a concentration that is effective against multidrug-resistant strains. Cohort studies have shown that the maintenance of a stable CD4 count despite virologic failure is accompanied by a lower risk of clinical progression. The use of foscarnet can be discussed case-by-case in the absence of another therapeutic possibility, to reduce the VL before introducing an ART that is not fully effective [72].

In these situations of multiple failure, therapeutic decisions should be taken at a multidisciplinary meeting. The opinion of a team experienced in the management of these patients is indispensable in situations where the therapeutic options seem limited (**AIII**).

After a change of ART because of virologic failure, it is necessary to measure VL and to rapidly evaluate the safety of the new treatment (after one month).

## Generic drugs and medical and economic considerations

These guidelines are likely to evolve rapidly as generic antiretrovirals, which will be markedly less expensive than reference drugs, gradually become available [73,74]. This advantage will be counterbalanced by the increased number of tablets when substituting generic-based combinations for brand single-tablet regimens, though no study showed a difference in virological efficacy and/or adherence in relation with the number of tablets as long as the treatment is taken once daily. For instance, a recent observational study showed that replacing Atripla^®^ by three generic tablets of each of its components in virologically suppressed patients was followed by maintained virologic suppression (Engsing, 20th CROI, Atlanta, 2013, Abstract 579). However, an increased daily number of tablets may not be well accepted by, and even can be deleterious to some patients.

## Conclusions

These guidelines recommend that any individual living with HIV should be treated with ART, irrespective of his/her CD4 cell counts and/or HIV plasma VL, including when CD4 cell count is above 500/mm^3^, which is in line with DHHS guidelines [75] but broadens the spectrum of indications for initiating antiretroviral therapy, as compared to other recent sets of guidelines (see Table 5). This recommendation was issued for the sake of patients as well as for promoting preventive measures. Cost issues were also taken into account in the selection of preferred first-line options as well as in suggesting that generic drugs should now also be considered for use in industrialized countries.

**Table 5:** Summary of “When to start” recommendations in patients living with HIV with asymptomatic disease, according to CD4 lymphocyte count, from five recent sets of Experts guidelines

	CD4 lymphocyte count (/mm^3^)			
	
Guidelines	<200	200–350	350–500	>500
British HIV Association, 2012	Start	Start	Consider^1^	Differ^1^
Department of Health and Human Services–USA, 2013	Start	Start	Start	Start
European AIDS Clinical Society, 2013	Start	Start	Start	Consider
World Health Organization, 2013	Start	Start	Start	Differ^2^
French guidelines, 2013	Start	Start	Start	Start

1 ARV treatment should be initiated in pregnant women; HCV and/or HBV co-infected patients; patients with HIVAN, HIV-associated neurocognitive disorders or cancer; and members of sero-discordant couples;

2 ARV treatment should be initiated in pregnant women; HBV co-infected patients with severe liver disease; tuberculosis co-infected patients; and members of sero-discordant couples.
